# Specific Targeting of Melanotic Cells with Peptide Ligated Photosensitizers for Photodynamic Therapy

**DOI:** 10.1038/s41598-017-15142-w

**Published:** 2017-11-16

**Authors:** Paul Lorenz Bigliardi, Bhimsen Rout, Aakanksha Pant, Viknish Krishnan-Kutty, Alex N. Eberle, Ramasamy Srinivas, Brendan Adrian Burkett, Mei Bigliardi-Qi

**Affiliations:** 1 Experimental Dermatology Group, Institute of Medical Biology, A*STAR, 8- Biomedical Grove, Singapore, Singapore; 2 Clinical Research Unit for Skin, Allergy & Regeneration, A*STAR, Singapore, Singapore; 3 University Medicine Cluster, National University Hospital Singapore, Singapore, Singapore; 4 Yong Loo Lin School of Medicine, NUS, Singapore, Singapore; 5 Organic Chemistry Division, Institute of Chemical and Engineering Sciences, A*STAR, Singapore, Singapore; 6 Department of Biomedicine, University of Basel, Basel, Switzerland

## Abstract

A strategy combining covalent conjugation of photosensitizers to a peptide ligand directed to the melanocortin 1 (MC1) receptor with the application of sequential LED light dosage at near-IR wavelengths was developed to achieve specific cytotoxicity to melanocytes and melanoma (MEL) with minimal collateral damage to surrounding cells such as keratinocytes (KER). The specific killing of melanotic cells by targeted photodynamic therapy (PDT) described in this study holds promise as a potentially effective adjuvant therapeutic method to control benign skin hyperpigmentation or superficial melanotic malignancy such as Lentigo Maligna Melanoma (LMM).

## Introduction

Photodynamic therapy (PDT) is an effective, non-invasive treatment for various cancerous and pre-malignant diseases which has gained increasing clinical interest in last few decades^1–4^. It is used in urology, gastroenterology and dermatology to treat superficial epithelial cancerous and pre-cancerous lesions. Applications in dermatology are particularly attractive because the photosensitizers can be topically delivered to the desired target without the patient experiencing systemic side effects. Furthermore, because skin is directly accessible to light, PDT offers opportunities to adjust the wavelengths and intensity of light delivered, as well as the photosensitizer to achieve the right combination to effectively target tissues or cells which show high uptake of that photosensitizer^5^.

One of the most widely employed targeted PDT methods currently employed for topical application in a clinical setting is δ-aminolevulinic acid (ALA), a precursor of proto-porphyrin IX (PPIX). Hyperactivated cells in tumors or infected cells show an increased intracellular metabolism of ALA and finally accumulation of PPIX in these cells. Therefore, ALA-PDT has been used successfully not only for the treatment of precancerous lesions (e.g. actinic keratosis, Bowen’s disease) and extensive superficial non-melanoma skin cancers (e.g. basal cell carcinoma)^6,7^ but also for other infectious or inflammatory diseases such as common, recalcitrant HPV infections, leishmaniasis, acne, and even cutaneous T-cell lymphomas. Although there is the advantage of specific accumulation and lack of interference of surplus residual ALA present near the targeting site during PDT, the quick systemic clearance and ineffectiveness towards melanomas and more advanced, invasive squamous and basal cell carcinomas remain amongst the concerns and limitations of this approach^8^.

Alternative approaches to treat more invasive and non-cutaneous cancers in gastroenterology and urology by PDT are based on the systemic use of haematoporphyrin derived photosensitizers such as verteporfin, temoporfin and 2-(1-hexyloxyethyl)-2-devinyl pyropheophorbide (HPPH). More recently these molecules have been tested for the treatment of malignant melanoma employing animal and human models^9^, where they were found to induce significant apoptosis, regression^6,10–15^, tumor growth arrest^15–17^ and tumor necrosis^18–20^ in both experimental and clinical studies. Slastnikova *et al*. used a modular nanotransporter and bacteriochlorin-p as PDT agent for targeted delivery to experimental mouse melanomas with excellent specificity^21,22^. Also, in human oncology, PDT was used as a primary effective therapy for choroidal melanoma^23,24^ and secondary therapy for ocular melanomas^25^. However, most of the clinical reports indicate limitations of this type of PDT, because of patient non-compliance and drop out of patients from the studies. This was due to partial remission and recurrences of the tumors, excruciating pain and massive collateral damages by non-targeted and non-specific phototoxic effects on both healthy and target tissues^20,26,27^.

Several studies have addressed the delivery of the haemoporphyrin derived photosensitizers^28–34^ to increase the specificity to target cells, including attachment to monoclonal antibodies^35–39^, peptides^40,41^, proteins^42,43^, saccharides^44^, aptamers^45^ or by nanomaterial encapsulation^46^. These methods are encumbered by inherent limitations such as large size, transport barriers^47,48^ and potential toxicity. Moreover, these studies have not included peptide ligands targeted against human melanoma/melanocytes; and none of these methods have addressed the issue of collateral damage that occur to the healthy surrounding cells which is a major concern for very early stage skin cancer/disorder, especially in delicate parts of the body (e.g. a mole on the face or forehead).

Recently, targeting of melanocytes and melanoma cells *in vitro* or melanotic lesions *in vivo* has been investigated using octapeptide derivatives of α-melanocyte-stimulating hormone (α-MSH) containing a chelator (DOTA) for radiometals such as ^111^indium, ^67/68^gallium or ^90^yttrium. This led to uptake and accumulation of radioactivity in melanomas of experimental animals^49,50^. MSH is the natural ligand of the melanocortin-1 receptor (MCR1), and octapeptide derivatives of α-MSH containing DOTA were shown to bind specifically to MC1R highly expressed on melanocytes and most malignant melanoma cells^51–53^. α-MSH also induces melanogenesis in melanocytes and melanoma cells but this process is slow^54^: melanins do not appear before 1–3 days after initial treatment with MSH peptides, i.e. at an interval when therapy sessions have long been terminated. The excellent targeting specificity of MSH octapeptide conjugates has been well documented^49^.

**Figure 1:**
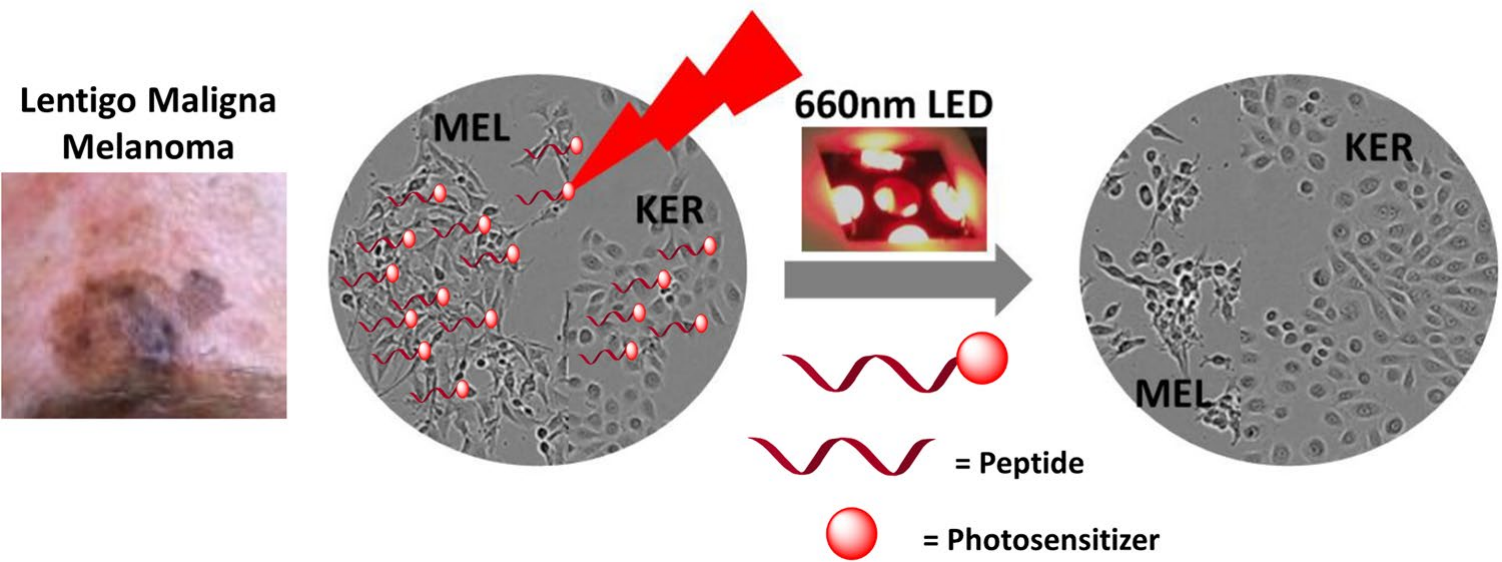
Left: Picture of a superficial lentigo maligna melanoma; Right: Schematic presentation of targeted killing of the melanotic melanoma cells with minimal damage to keratinocyte using a peptide ligated photosensitizer at 660 nm LED-light.

In this study, we describe the development and biological properties of an MSH octapeptide conjugated to a non-selective photosensitizer for specific targeting of melanoma cells, via binding to MC1R followed by receptor-mediated internalization. Utilizing highly precise and tandem near-IR LED light (which has ability to penetrate much deeper in the skin), the cytotoxicity to the target melanoma cells is achieved with minimal collateral damage to the peripheral keratinocytes (for pictorial presentation, see Fig. 1). This could lead to a potentially more effective, non-toxic, and sophisticated technology for specific cytotoxicity (i.e. controlled pigmentation) with minimum collateral damage.

## Result and Discussion

**Figure 2:**
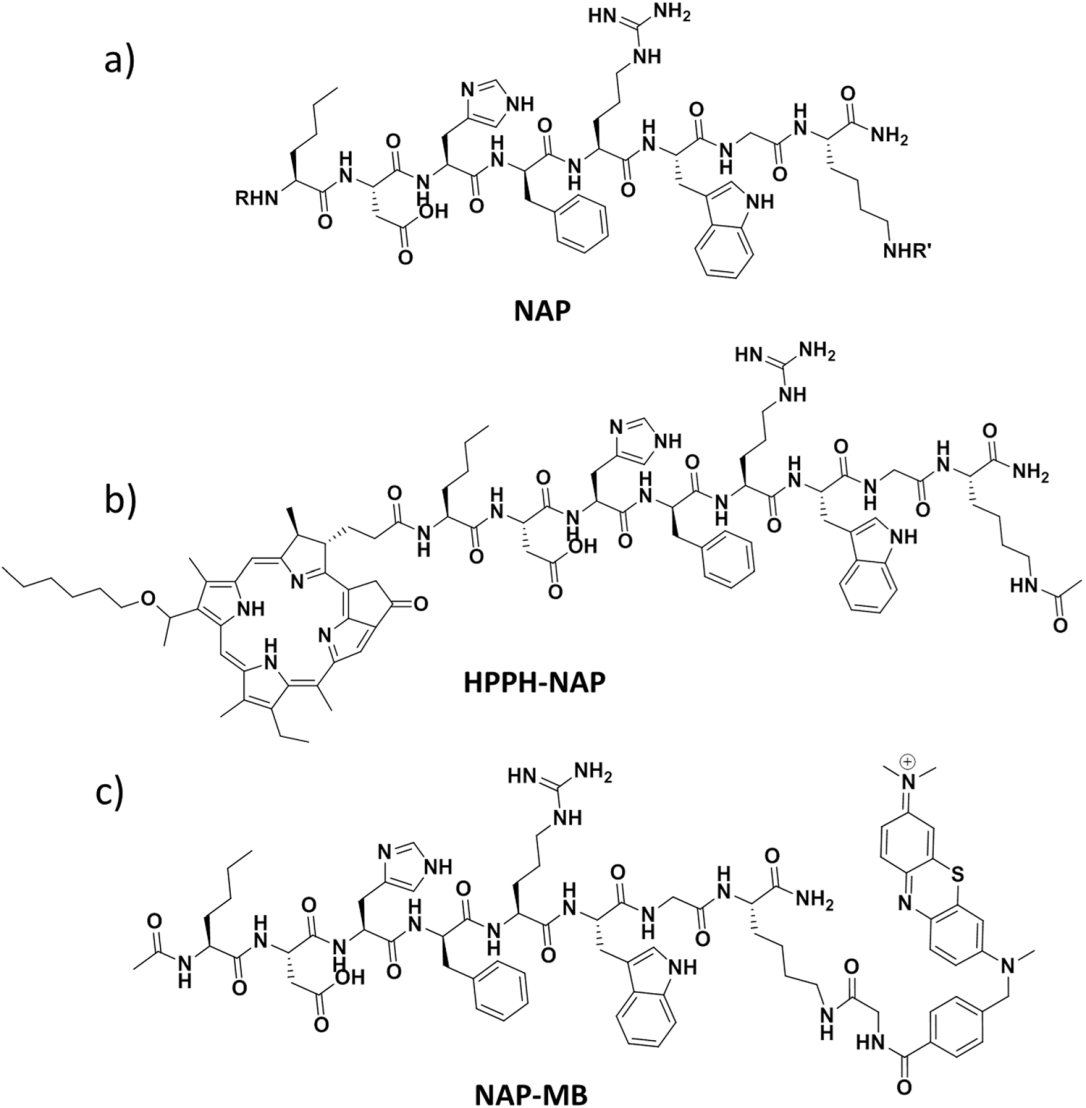
Structure of the MC1 receptor peptide ligand NAP (**a**) where R = H/Ac, R′ = Ac/H; Structure of its photosensitizer derivatives HPPH-NAP (**b**) where R = HPPH, R′ = Ac, and NAP-MB (**c**) where R = Ac, R′ = MB.

The (4–11) octapeptide sequence of α-MSH, appropriately modified, has been shown to be the best choice in replacing the relatively instable tridecapeptide sequence of α-MSH for *in vitro* and *in vivo* studies. We selected NAP-amide^49^ (Fig. 2) as targeting peptide, as this molecule is active in the sub-nanomolar concentration range and has been shown by different laboratories to be an excellent compound for melanoma targeting^52^. Among various photosensitizers, HPPH and methylene blue (MB) were chosen for covalent conjugation with NAP. These photosensitizers absorb at 660 nm, i.e. in the spectral range of 650–800 nm that is considered applicable for PDT of melanotic melanoma (a very small peak of melanin absorption exist beyond the 650 nm wavelength but is regarded unproblematic)^55,56^. HPPH has a higher molecular weight and is more hydrophobic than the charge-bearing MB. Molecular size, aqueous solubility and polarity of the final synthetic products are important factors for future intra-cutaneous delivery, factors that ultimately determine the ability of the conjugates for transport through the corneal layer, live epidermis and upper part of dermis where potential target cells are located. Moreover, these properties also affect cellular uptake, ROS formation, cytotoxicity and finally clinical efficacy^57,58^.

To this end, the free N-terminal amine of NAP was covalently attached to the acid group of HPPH using pyBOP and DIPEA as coupling agent and base respectively; the reaction produced HPPH-NAP (Fig. 2b) confirmed by mass spectrometry. In contrast, MB was attached to the lysine side-chain of NAP (Fig. 2c): the aromatic amine group of Azure B was first modified with the tert-butyl derivative of 4-bromomethyl pheny lcarboxylic acid by warming to 47 °C and using basic conditions. Subsequently, TFA-mediated deprotection of the tert-butyl group generated the free acid, followed by *in-situ* conjugation with C-terminal lysine of NAP using pivaloyl chloride, thus furnishing NAP-MB (see supporting information, Scheme S2b-e). No over-reacted products (i.e., additional photosensitizer coupled products) was observed in mass spectroscopy indicates highly reactive nature of primary amine of the lysine that is present on the end of the peptide (i.e., less hindered) over other nitrogen present on imidazole ring and arginine moiety. In addition, the NAP-MB conjugate, after purification using two different gradients (see supporting info for synthetic procedure) has shown the correct mass in ESI spectrometry but no sign of peak splitting, broadening and no additional peak in analytical HPLC which indicates that MB was conjugated exclusively through the highly reactive amine of lysine.

Another advantage regarding their photo-chemical properties is that HPPH and MB have common absorption peaks at far-visible/near-IR wavelength (i.e. 660 nm) but different ROS generating abilities upon excitation at same wavelength. This allows a high-throughput PDT screening with one and the same light source, using a precisely defined wavelength with comparable light quantities. In addition, the near-IR wavelengths of 660 nm penetrate deeply into the skin, down to the subcutaneous fat tissue whereas shorter wavelengths do not^59^. We developed a patented LED device capable of delivering standardized light quantities at specific wavelengths (+/− 10 nm) on cells grown on tissue cultured plates^60^. The system was incorporated into the Incucyte-FTR (Essen Bioscience), which is a fully automated device that images cells in real time thus enabling the progressive study of cell morphology, cell density and cell proliferation over time. The delivery of well-defined and localized near-IR irradiation to the cells containing the photosensitizer will result in production of ROS damaging the membranes of targeted cells and their organelles through oxidation of lipids and proteins and resulting in apoptosis, necrosis or cell cycle arrest in these cells^57^. Hence, the novel synthetic molecules were screened *in vitro* using various skin cell types, such as mouse melanoma cells (B16-F10), human melanoma cells (FM55), primary human melanocytes and immortalised human skin keratinocytes (N/TERTs) exposed to 660 nm inducing in target cells in short term specific cytotoxicity and/or long term skin pigmentation.

Literature precedence^54,61^ revealed that murine melanoma B16-F10 cells have higher densities of MC1 receptors and higher basal levels of melanin compared to most of the human melanoma cell lines (FM55) and primary human melanocytes. Murine B16-F10 cell line and human FM55 melanoma cells were used as models to study binding properties and functional efficacies of native peptides (α-MSH, NAP) and various peptide-photosensitizer constructs by measuring the amount of extra- and intracellular melanin production.

**Figure 3:**
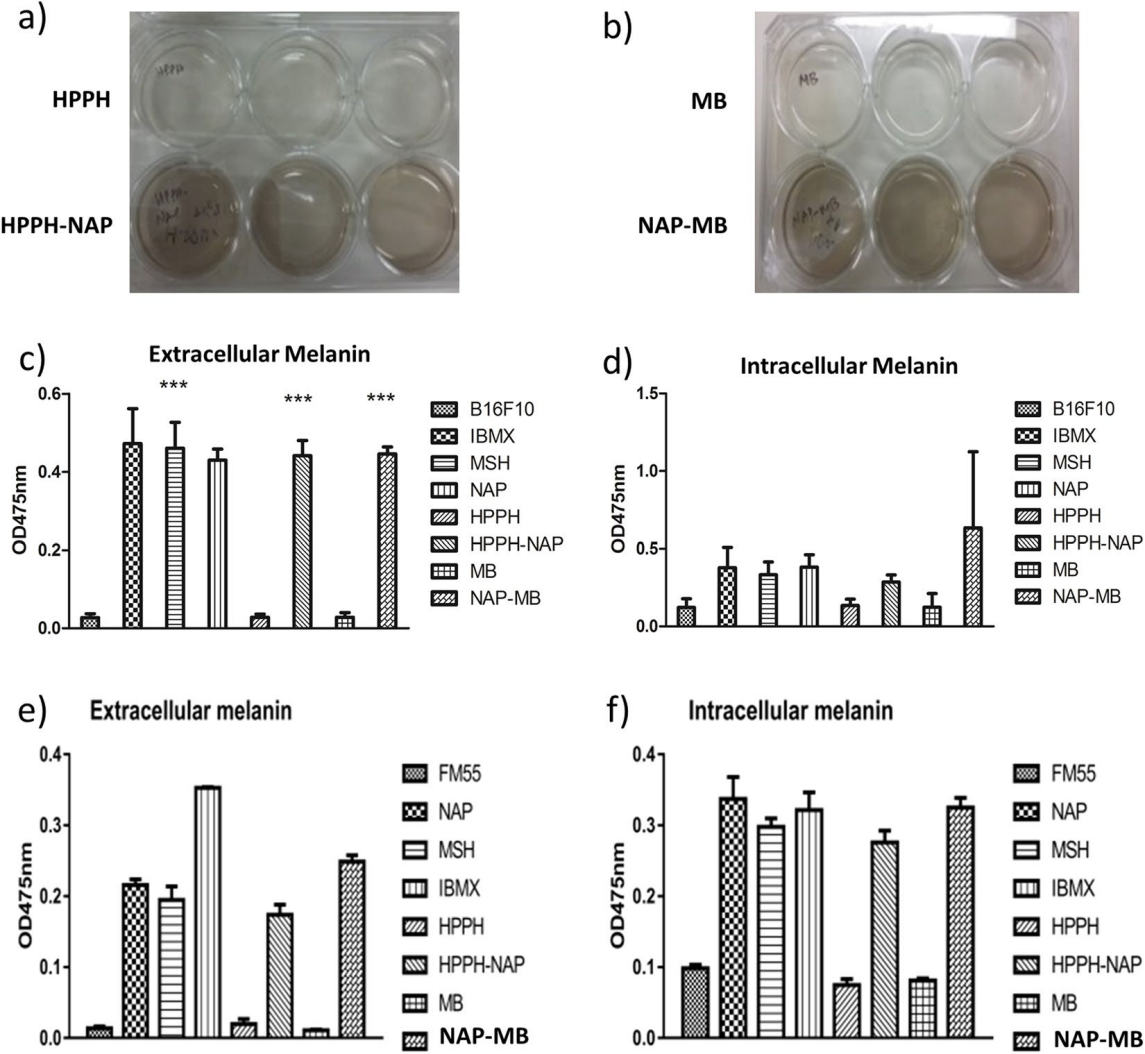
Visual color change upon addition of HPPH and HPPH-NAP (**a**) and MB and NAP-MB (**b**) at 1 µM concentration to B16-F10 mouse melanoma cells. Quantitative determination of extracellular and intracellular melanin production by α-MSH (10 nM), IBMX (50 µM), NAP (1 µM), HPPH-NAP (1 µM), MB (1 µM) and NAP-MB (1 µM), to B16-F10 (**c**,**d**) mouse melanoma and FM55 (**e**,**f**) human melanoma cell lines. Optical density (OD) of melanin was measured at 475 nm after 72 h. Significance^φ^: ***p ≤ 0.001.

As shown in Fig. 3, photosensitizers alone (i.e. HPPH, MB) did not induce any melanin production in B16F10 cells, whereas all peptides (i.e. MSH, NAP) and their photosensitizer derivatives (i.e. HPPH-NAP, NAP-MB) were able to generate within 72 h considerable amounts of extra- and intracellular melanin. The long duration of melanin production by the peptides and photosensitizer derivatives was comparable to the positive control 3-isobutyl-1-methyl xanthine (IBMX). Substantial production of extracellular as well as intracellular melanin in both murine B16-F10 cells (Fig. 3c,d) and FM55 human melanoma cells (Fig. 3e,f) indicate targeting and binding nature of the different peptides and their photosensitizer derivatives to the MC1 receptor at sub-micromolar concentrations. The increase in cellular uptake is due to the targeting ability of the peptides with its balanced lipophilicity/hydrophilicity nature as demonstrated with radiolabelled DOTA ligands which supports the melanin production results^62,63^: Significant melanin production using α-MSH with B16 melanoma cell line is observed at 72 h, whereas negligible amount of melanin was produced in first 36–48 h^62^. This safe time-window of first 48 h without considerable melanin production is important in our study, because this allowed us to do light induced cytotoxicity measurement within 24 h without much interference by melanin, a stable protein-complex with a wide absorption spectrum from 400–650 nm. In addition, it has been reported in literature that the resistance of malignant melanotic lesions to PDT was due to the production of the melanin, which competes with photosensitizers for photons, resulting in inefficient phototoxicity^55^. If the melanotic cells are killed during the first 48 h, this problem does not occur. Therefore, our future toxicity assays are demonstrated on the first 24 h.

**Figure 4:**
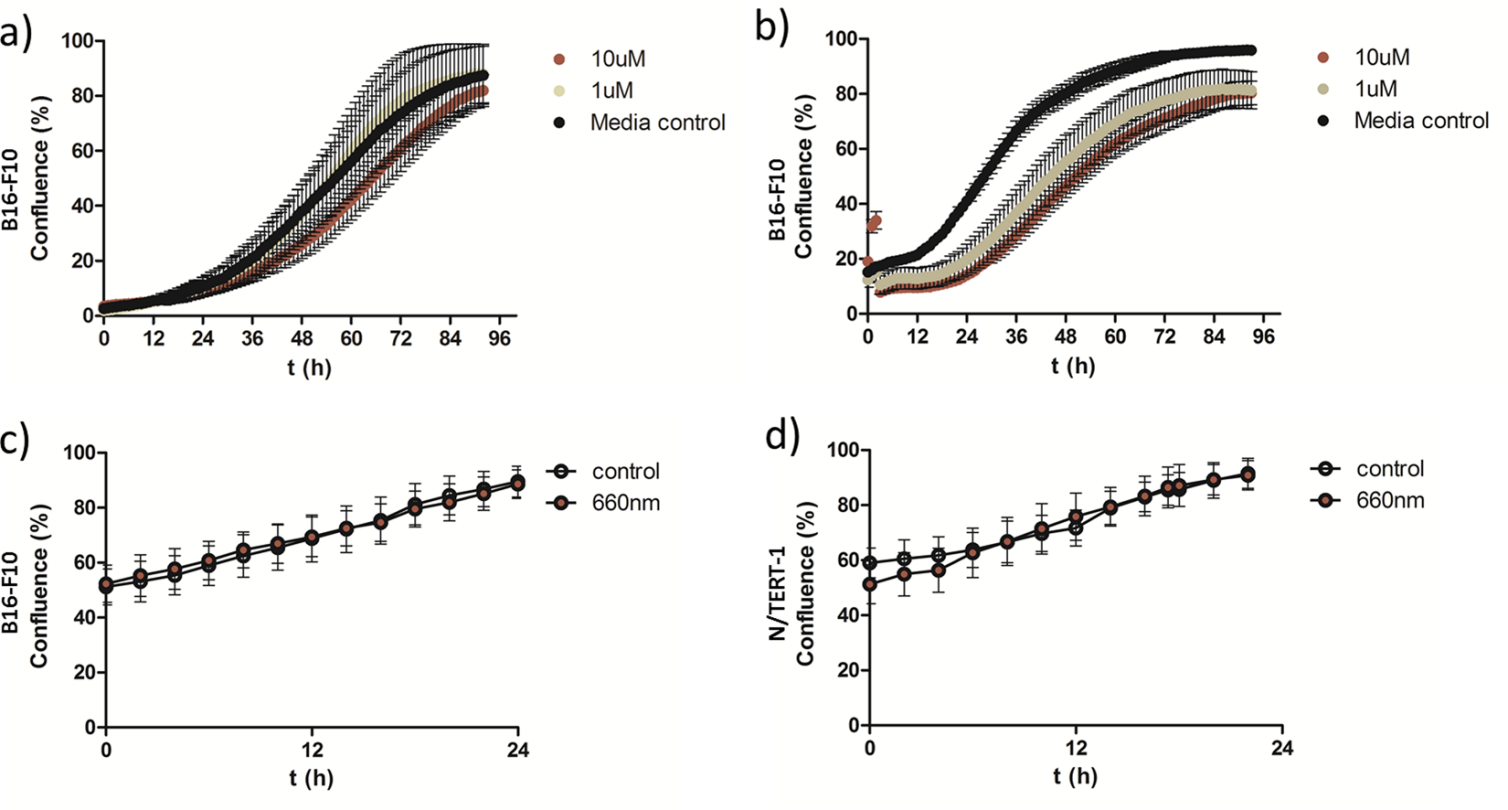
Effect of (**a**) HPPH-NAP and (**b**) NAP-MB on B16-F10 cell proliferation^⊥^ (no light exposure). The effect of 660 nm light on survival/proliferation^⊥^ of (**c**) B16-F10 and (**d**) N/TERT-1 cells.

In clinical settings, all medically used photosensitizers should be well-tested to be non-toxic under dark conditions and the wavelength of light used during therapy must also be non-toxic to skin cells which are the primary requirements for PDT. Although, HPPH-NAP and NAP-MB bind to MC1-receptor at low micromolar concentrations, it was important to investigate cell proliferation and toxicity in the dark without light stimulation. A cell proliferation assay was performed in dark conditions on B16-F10 cells in a dose response of HPPH-NAP from selected concentrations of 10 µM to 100 nM (see supporting information, Figure S1). The non-toxic nature of HPPH-NAP at 10 µM and 1 µM for prolonged period of time, i.e. 96 h (see Fig. 4a), demonstrates its suitability as a potential therapeutic candidate. In addition, the light used for this study, i.e. 660 nm with an intensity of 0.1 mW/cm^2^, did not affect the proliferation of B16-F10 cells and N/TERT-1 cells (Fig. 4c,d) as cells showed healthy morphology (Figure S3).

**Figure 5:**
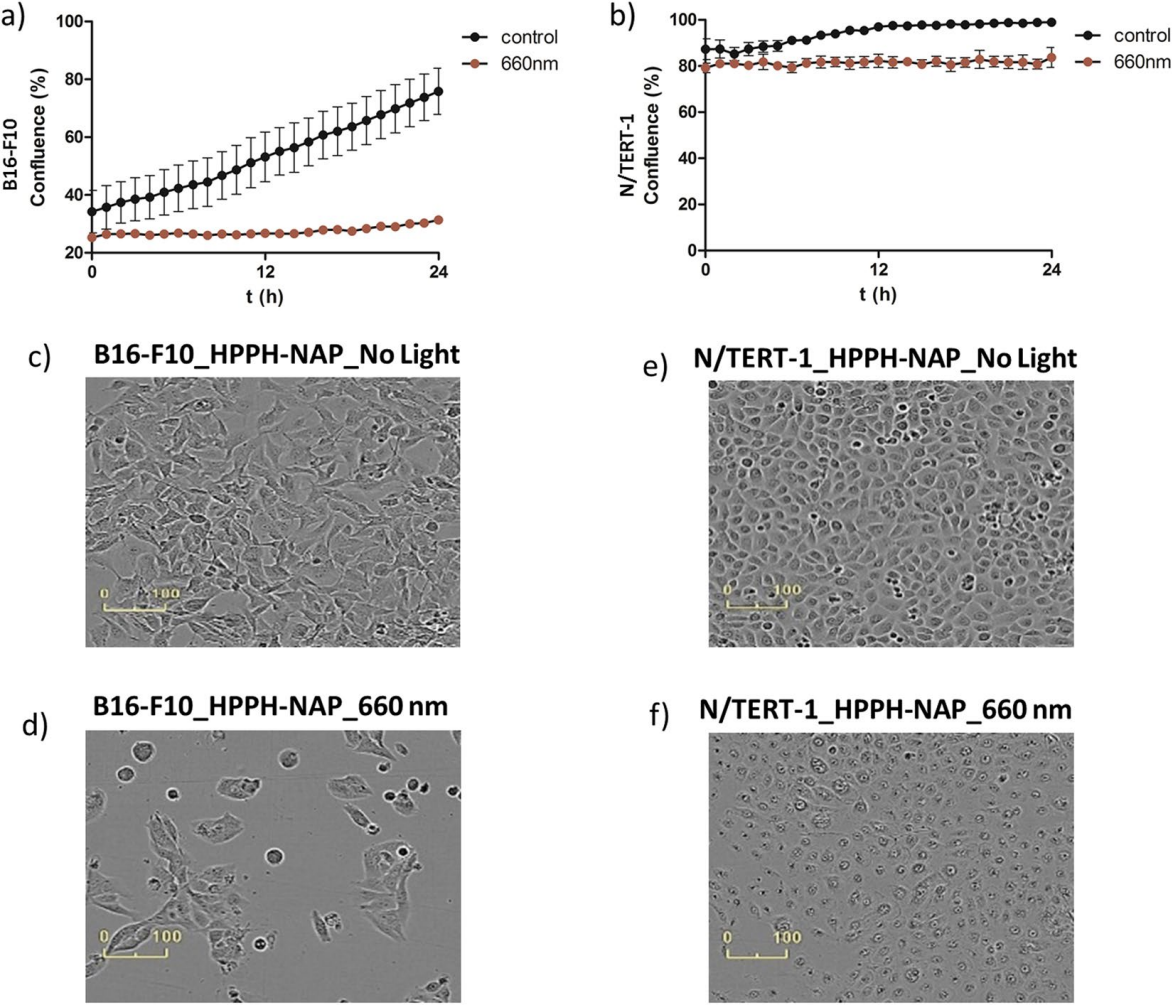
Cell proliferation^⊥^ curve of B16-F10 (**a**) and N/TERT-1 (**b**) cells treated with HPPH-NAP (5 µM) and 660 nm light for 24 h. Corresponding cell growth image after 24 h for B16-F10: (**c**) no light, (**d**) 660 nm light and for N/TERT-1: (**e**) no light, (**f**) 660 nm light irradiation.

By irradiating HPPH (5 μM) with 0.10 mW/cm^2^ of LED light for 48 h continuously, cytotoxicity was observed at both 627 nm and 660 nm (Figure S2). The cell proliferation continues after stopping the light source at 627 nm but not at 660 nm. In the case of HPPH-NAP (5 μM), more cytotoxic effect and drastic reduction of B16-F10 melanoma cells were noticed at 660 nm light with an intensity of 0.1 mW/cm^2^ continuously over 24 h. Incucyte images were taken at an interval of one hour and changes in cell densities (i.e. confluency) were measured as shown in Fig. 5a. Reduction in cell growth and proliferation defect was observed for the first 24 h exposure of near-IR LED light in comparison to the control. The quick termination of cell growth was due to the combination of HPPH-NAP and light. The sole effect of light was excluded by the exponential proliferation and lack of growth defect, by exposing melanoma cells to the light of 660 nm without incubating with HPPH-NAP (Fig. 4c,d). Incucyte images taken after 24 h were used to compare the morphologies of cells studied between HPPH-NAP with light and HPPH-NAP without light. Morphologically damaged, rounded B16-F10 melanoma cells were observed after exposure to 5 µM HPPH-NAP and 0.1 mW/cm^2^ light at 660 nm (Fig. 5d). In contrast, melanoma cells just cultured for 24 h with 5 µM HPPH-NAP and no light exposure were growing well with healthy morphology (Fig. 5c).

Achieving appreciable cytotoxic effects on melanoma cells, it was interesting to test the collateral damage using an immortalized skin keratinocyte line, N/TERT-1 at higher confluency to better mimic the physiological conditions of the epidermal melanin unit system in the skin where keratinocytes are present in much higher number than melanocytes^64^. However, 5 µM HPPH-NAP was found to inhibit N/TERT-1 cell proliferation (Fig. 5b, brown curve) with appearance of rounded and granular cell morphology (Fig. 5f) after exposure to 0.1 mW/cm^2^ light at 660 nm for 24 h. This is in contrast to control conditions with cells exposed to 5 µM HPPH-NAP in Fig. 5e and kept in dark for 24 h (see also Fig. 5b, black curve), where cells proliferate to 100% confluency and is therefore perhaps not optimal for specific, targeted PDT.

**Figure 6:**
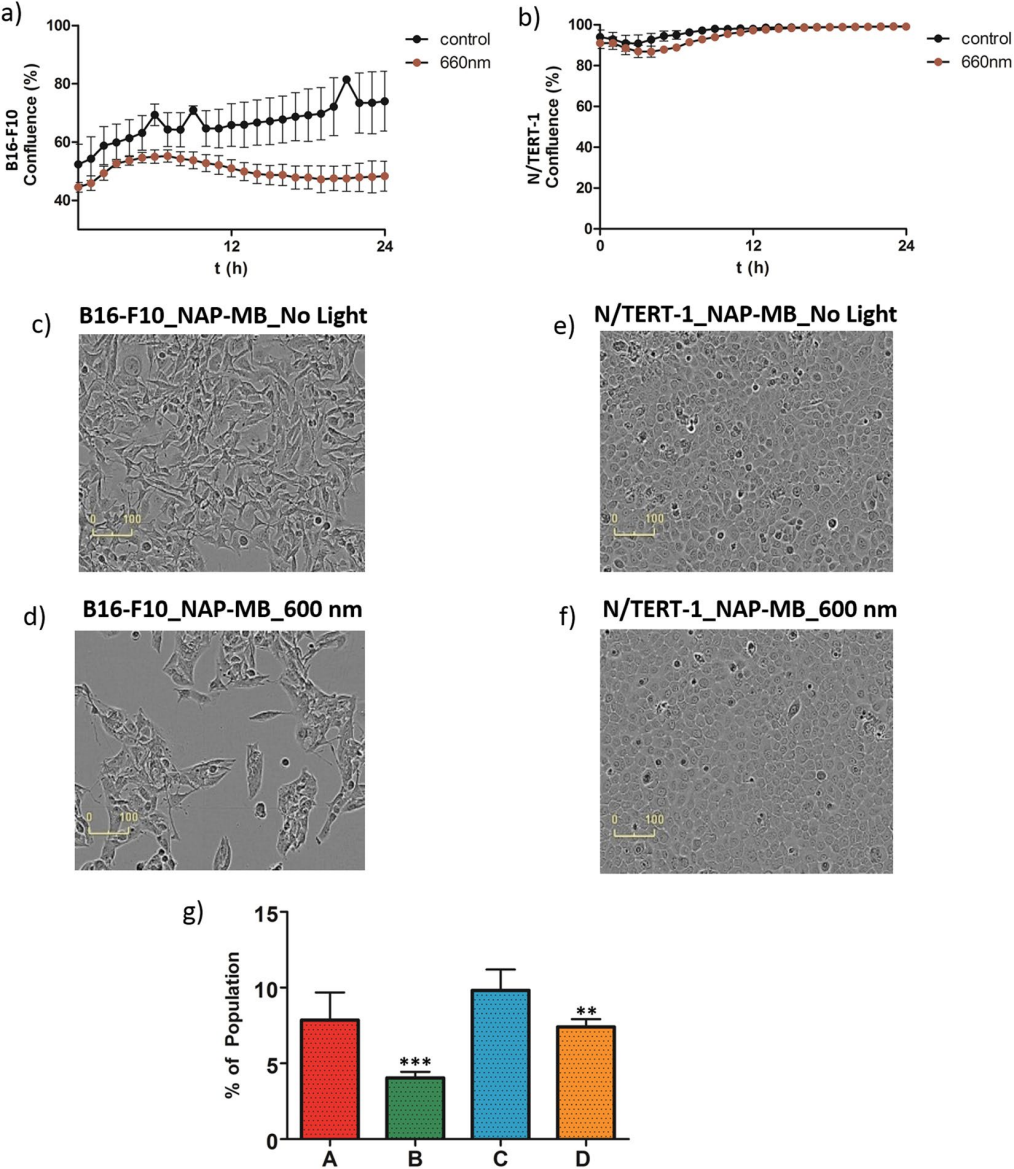
Cell proliferation^⊥^ curve of B16-F10 (**a**) and N/TERT-1 (**b**) cells treated with NAP-MB (10 µM) and 660 nm light for 24 h. Corresponding cell growth image for B16-F10 after 24 h: (**c**) no light, (**d**) 660 nm light and for N/TERT-1: (**e**) no light, (**f**) 660 nm light. (**g**) Competition assay of NAP-MB in presence of excess unconjugated NAP where percentage of NAP-MB bound cell population presented upon incubation of A) 1 µM NAP-MB at RT, B) 1 µMNAP-MB and 250 µM NAP at RT, C) 1 µM NAP-MB at 4 °C, D) 1µMNAP-MB and 250 µM NAP at 4 °C.

In the search for a photosensitizer with less collateral damage to keratinocytes we expanded our experiments to methylene blue (MB), another clinically used photosensitizer. The effect of C-terminal lysine linked NAP-MB (for structure, see Fig. 1c) on murine B16-F10 melanoma cell proliferation in dark was performed and was found to be non-toxic both at 10 µM and 1 µM concentration for prolonged period of time (Fig. 4b). The minor difference in proliferation was due to slight difference in starting cell densities. The B16-F10 melanoma cells incubated with NAP-MB at 10 µM were irradiated for 24 h with 660 nm light with an intensity of 0.1 mW/cm^2^ resulting in high cytotoxicity and reduction in cell densities (Fig. 6a). Proliferation defects and unhealthy cellular morphology was observed at 24 h for melanoma cells treated with NAP-MB under 660 nm light (Fig. 6d) compared to the no light control (Fig. 6c). However, significantly healthy morphology (Fig. 6f) and cell proliferation to 100% confluency (Fig. 6b, brown curve) was observed for N/TERT-1 keratinocytes incubated with NAP-MB even after 24 h of light exposure at 660 nm. Healthy cell morphology (Fig. 6f) was noticed similar to cells that were grown under no light conditions (Fig. 6e) indicating reduced collateral damage. As 10 µM NAP-MB conjugate is more specific and working better than 5 µM HPPH-NAP resulting in higher cytotoxicity to melanoma cells with less collateral damage to other peripheral skin cells and therefore, NAP-MB was taken further to demonstrate competition assay and quantitative Sulforhodamine based (SRB) cytotoxicity assay.

In Fig. 6g, specific binding of NAP-MB to B16-F10 mouse melanoma cells were demonstrated by competition assay using unconjugated peptide (NAP) at 4 °C and at room temperature. Quantitatively, near-equal population of cells containing MC1-receptor bound to NAP-MB at two different temperatures (A, C) were observed. The fifty percent reduction in the NAP-MB-bound-population at room temperature (B) in presence of 250 fold excess of unconjugated NAP indicates that both NAP and NAP-MB were competing for the same MC1-receptor target, and the higher concentration of the NAP binds significantly to the MC1-receptor resulting the substantial reduction in population at room temperature. The competition between NAP-MB and excess of unconjugated NAP were found sluggish at 4 °C as found by 25% reduction in the population (D). Probably, the receptor trafficking is much less at 4 °C and results less internalisation, which may explain the observed difference.

All the incucyte experiments were done by exposing the target cells to the photosensitizer for 4 h, then washing it away and irradiating the cells with 660 nm light at 0.1 mW/cm^2^ over 24 h. However, under natural therapeutic conditions, the photosensitizer could be present in the tissue for more than 24 h, particularly with a known renal clearance taking at least 24 h. A variation in the cytotoxicity assays were done by leaving the 1 µM NAP-MB for longer duration i.e. 24 h during the entire light exposure, after a 4 h pre-incubation in the cell medium by measuring cellular protein contents and therefore, cell proliferation/toxicology by colorimetric assay of Sulforhodamine B.

**Figure 7:**
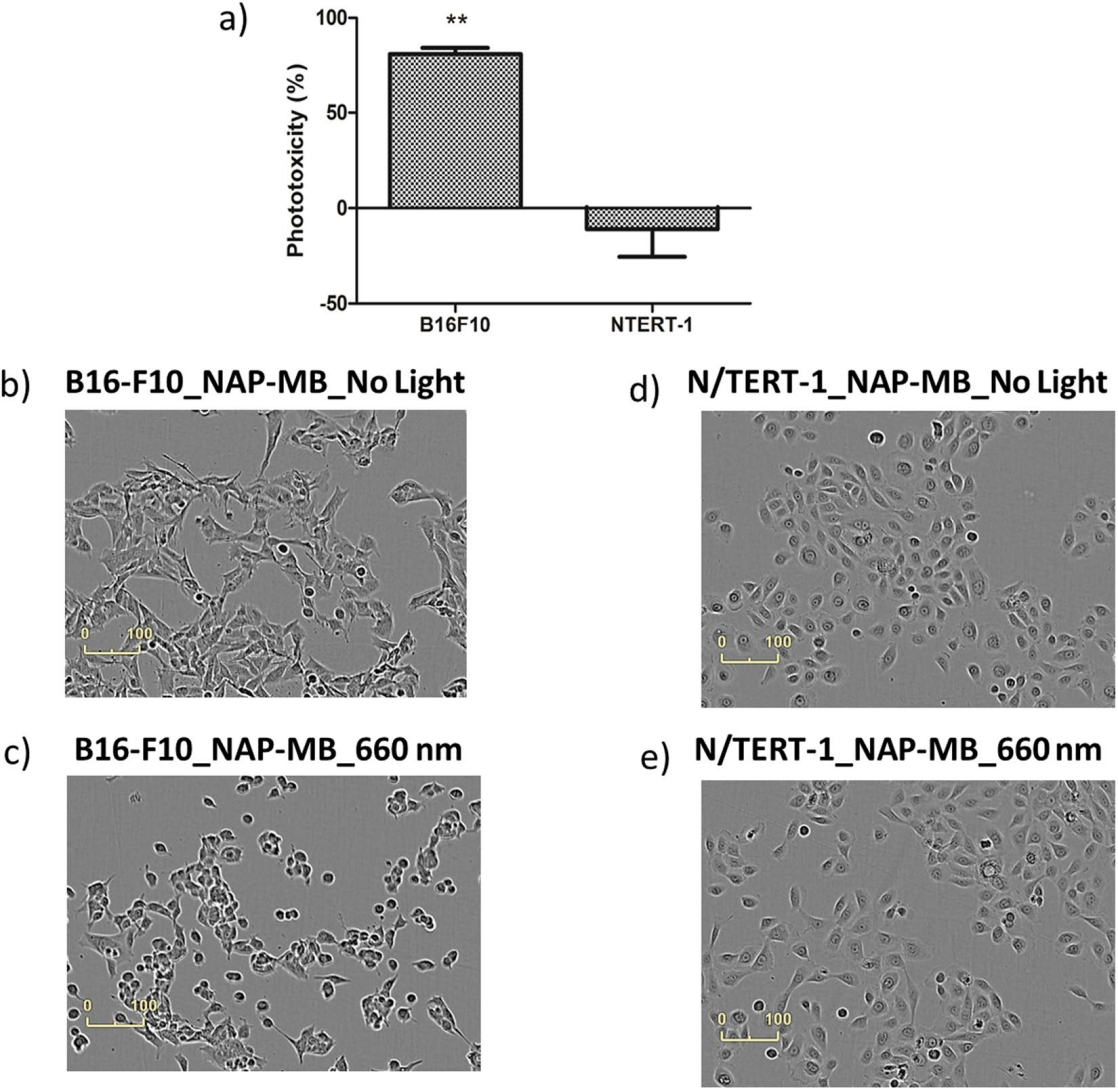
(**a**) Phototoxicity (%) of NAP-MB (1 µM) with B16-F10 and N/TERT-1 measured by SRB colorimetric assay (Significance^φ^: **p ≤ 0.01). Incucyte cell growth image for B16-F10 after 24 h: (**b**) no light, (**c**) 660 nm light and for N/TERT-1: (**d**) no light, (**e**) 660 nm light.

Figure 7a shows the quantitative cytotoxicity in the SRB assays at 1 µM NAP-MB using melanoma cells (B16-F10) and peripheral keratinocyte cells (N/TERT-1). As seen previously (in Fig. 6a–f) with incucyte evaluation, there was again morphologically and by colorimetry a significant cytotoxic effect of NAP-MB with light exposure on melanoma cells, but not to N/TERT keratinocytes. The longer duration of cells exposed to NAP-MB allowed to reduce the amount of photoactive peptide by 10-fold. The results from the SRB assay are supported by the morphological images of the cell cultures taken after 24 h light exposure with remarkable signs of damage i.e. cells are rounded and deforming instead of elongated cell morphology was observed in B16-F10 melanoma cells (Fig. 7b,c) with no morphological indication of cell damage for N/TERT-1 keratinocyte (Fig. 7d,e).

**Figure 8:**
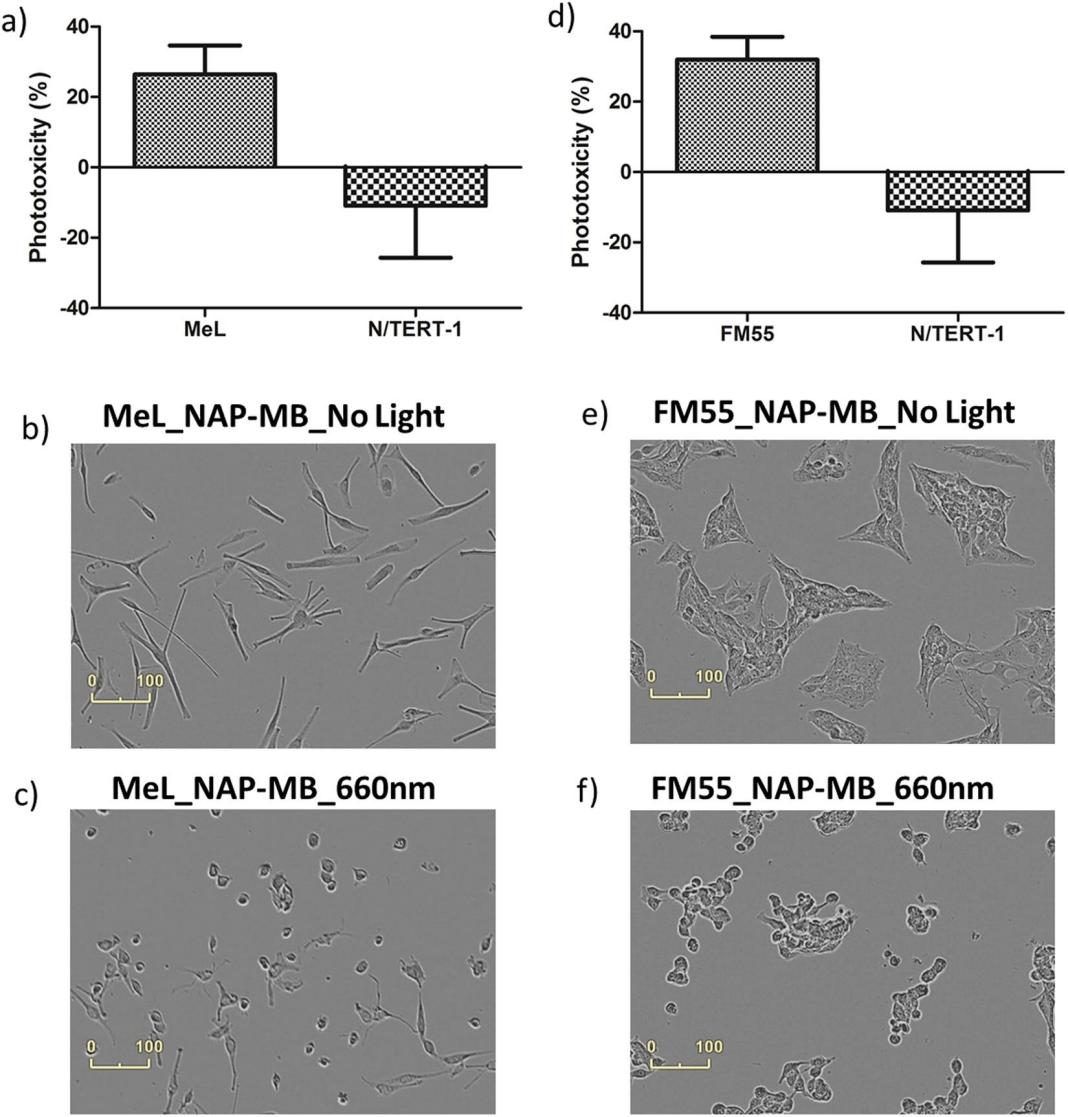
Phototoxicity (%) of NAP-MB (1 µM) with (**a**) MeL & N-TERT-1 and (**d**) FM55 & N/TERT-1 measured by SRB colorimetric assay. Incucyte cell growth image for MeL after 24 h: (**b**) no light, (**c**) 660 nm light and for FM55: (**e**) no light, (**f**) 660 nm light.

Furthermore, the cytotoxic effect of the peptide-photosensitizer conjugate, NAP-MB was tested on human primary melanocytes (MeL) and human melanoma cells (FM55) upon exposing to 660 nm light. Figure 8a and d represent quantitative phototoxicity of NAP-MB on melanocytes and melanoma FM55 respectively. The results indicate appreciable toxicity for both these cell types when compared to keratinocytes, N/TERT-1. However, Mouse melanoma cells, B16-F10 show higher levels of toxicity compared to human melanoma FM55 cells and particularly primary human melanocytes, as these cells have reduced amounts of MC1 receptors compared to mouse cell lines with approximately 10-fold more MC-1 receptor expression^64,65^. Nevertheless, morphological images of the cell cultures taken after 24 h light exposure support the SRB cytotoxicity data with clear cell damage to melanocytes and FM55 cells, but none to keratinocyte.

## Conclusion

Various experimental designs and different quantitative and qualitative assays demonstrate that N/TERT skin cells were not affected by NAP-MB at high (85–95%; Fig. 6) and lower (35–40%; Figs 7 and 8) cell confluency. However, the melanocytes and melanoma cells were severely affected by the photosensitizer in combination with light exposure suggesting an important reduction of collateral damage by specific and targeted killing of melanoma cells and melanocytes. Hence, a strategy was developed by covalent conjugation of photosensitizers to a MC1 receptor-targeting peptide. This method successfully achieved specific cytotoxicity to the melanoma cells under precise LED light dosage at near-IR wavelength with minimum collateral damage to peripheral skin cells. Further studies using formulated peptide-MB conjugates on in-house 3D-organotypic cultured^66^ human melanoma skin models under light would be useful to better understand how these compounds could be deployed in a clinical setting^67^. The concept of photodynamic therapy has as well the benefit to irradiate very targeted only the areas that have to be treated and the surrounding skin can be covered and there will be no damage to the pigmented cells. This technology aims to be the preferred choice over radiation and have clinical potential for treatment of superficial benign and malign melanotic lesions, particularly if they are of great surface and/or on delicate parts of the body (e.g. face, fore-head) or if the patient cannot undergo an operation (e.g. old age). The specific killing of melanoma cells that was achieved in this study is an effective subsidiary therapeutic method to control skin pigmentation which potentially can be used for cosmetic application. The near-IR wavelength light used in this study has potential to address the issue of much deeper skin tissue disorder (e.g. subcutaneous melanoma). The newly designed photosensitive peptide can be used for very targeted and personalized treatments of benign and malign pigmented lesions^63^.

## Methods

### Cell culture

B16-F10 mouse melanoma cells, HBL and FM55 human melanoma cells were maintained in DMEM media supplemented with 10% fetal bovine serum and 1% penicillin-streptomycin. Primary human melanocytes, MeL were maintained in medium 254 supplemented with human melanocyte growth supplement 2, PMA free and 1% penicillin-streptomycin. To prevent interference of light absorption by the media, DMEM media without phenol red was used in near-IR light mediated photodynamic therapy. N/TERT-1 human keratinocytes were maintained in K-SFM media supplemented with 0.4 mM calcium, 0.2 ng/ml epidermal growth factor, 25 μg/ml bovine pituitary extract and 1% penicillin-streptomycin.

### Melanin assay (general procedure)

B16-F10 mouse melanoma cells or human mid-pigmented melanoma cells FM55 were seeded in Nunc 6-well tissue culture plates using DMEM medium without phenol red. After overnight incubation of the cells at 37 °C, various photosensitizers (1 µM) or peptide conjugated photosensitizers (1 µM) were added at room temperature and the cells were incubated at 37 °C for 72 h. After three days of incubation, supernatants of cell (200 µL) were pipetted out from Nunc 6-well tissue culture plates as extracellular melanin pigment and three concurrent absorption measurements were taken at 475 nm using a Spectra Max MS microplate/cuvette reader (Fig. 3c,e). Adherent cells in the Nunc 6-well plates were detached with 0.02% EDTA and centrifuged at 2000 rpm for 3 min. The cell pellets were dissolved in 1 M NaOH (200 µL) and then heated at 75 °C for 5 min in order to lyse the cells to release intracellular melanin pigments and subsequently cooled down to room temperature. Three concurrent absorption measurements were taken at 475 nm using a Spectra Max MS microplate/cuvette reader (Fig. 3d,f). 10 nM α-MSH (melanocyte stimulating hormone) or 50 µM IBMX (3-isobutyl-1- methyl xanthine) were used as positive control because these are commonly known as melanin inducers.

### Cell proliferation assay (general procedure)

B16-F10 murine melanoma cells were seeded at a density of 3000–4000 cells per well in 96-well black-view Perkin-Elmer plates using DMEM medium without phenol red. After an overnight incubation at 37 °C, the medium was aspirated from the wells and varying concentrations of synthetic peptide-photosensitizer constructs diluted in DMEM medium were added to the cells. The cells were incubated in dark at 37 °C for 4 h and then washed twice with 1X PBS buffer (200 µL). Fresh medium (300 µL) was added to the wells before placing the plates in the Incucyte ZOOM live cell imaging machine to capture the images after every hour. Percentage of confluence was measured over a time period of 24–72 h. For skin keratinocyte cell line N/TERT-1, the starting confluence was 75–85% of cell density for the initial light experiments. The higher cell density was used to mimic the physiological condition in the body, where keratinocytes are present in much larger numbers (30:1) than melanocytes.

### Flow cytometry

Adherent B16F10 melanoma cells were subjected to dissociation using TrypLE (Gibco, Thermo Fisher Scientific Inc., Singapore) and washed with Phosphate Buffered Saline (PBS) before re-suspension in buffer (PBS + 1% bovine serum albumin). NAP and NAP-MB treatment was carried out at a density of approximately 100,000 cells per ml in buffer on room temperature (RT) or 4 °C for 30 min with the indicated concentrations before acquisition on the BD LSR Fortessa™ (BD Biosciences, San Jose, CA, U.S.A.). Signal from the methylene blue dye was detected using the 647 nm excitation and BP660nm emission filter. Samples were prepared and analyzed in triplicates or more. Subsequent population and data analysis was carried out using FlowJo_V10 software (FlowJo LLC, Ashland, OR, USA).

### Sulforhodamine (SRB) Phototoxicity assay

Mouse melanoma B16-F10 cells, primary human melanocytes MeL, mid-pigmented human melanoma FM55 cells and human keratinocytes N/TERT-1 were seeded at cell densities to achieve similar confluence after overnight incubation to perform SRB cytotoxicity assay. Cells were incubated with 1 µM of NAP- MB, and left in the medium with 0.10 mW/cm^2^ of 660 nm light for 24 h. After the end of the experiment, cold 10% trichloroacetic acid (100 µL) was added to the wells. Upon one hour incubation at 4 °C, the cells were washed five times with water and air dried. Further, SRB in 1% acetic acid (100 μl of 0.057% (wt/vol) SRB solution) was added to each well. After 30 min of incubation at room temperature, cells were washed thrice with 1% acetic acid and air dried. 10 mM Tris-base (200 µL) was added to solubilize the dye with gentle agitation. Optical density was measured at 510 nm wave-length. The percentage of phototoxicity was calculated using the following equation:

$$Phototoxicity\,( \% )=1\,-\,\frac{({{\rm{OD}}}_{{\rm{Treatment}}}\,-\,{{\rm{OD}}}_{{\rm{Blank}}})}{({{\rm{OD}}}_{{\rm{Control}}}\,-\,{{\rm{OD}}}_{{\rm{Blank}}})}$$

where OD_Treatment_ represents optical density measured for cells treated with peptide-photosensitizer constructs and light at 660 nm. OD_Blank_ represents optical density measured for media. OD_Control_ denotes optical density measured for cells treated with peptide-photosensitizer constructs.

### Incucyte LED light setup and imaging

This combinatorial equipment comprises light irradiation system with multiple wavelengths LED (From UVA to near IR) assembled with well-established incucyte that contains incubator and light microscope. The instrument is equipped with state-of-the-art feedback mechanism that can interpret the actual amount of energy being delivered to the samples. In current study, we have used only 660 nm LED light source. The energy of light irradiation was 0.10 mW/cm^2^ produced continuously and was found stable for a period of 24–72 h. Incucyte light microscope was used to capture images of the cell growth at every hour or two, from which percentage confluence (i.e. cell densities) were calculated.

### Statistical analysis

B16-F10 and FM55 melanin assays are represented as the average of three independent experiments expressed as Mean ± SEM. Differences between various groups were calculated using one way ANOVA post Bonferroni’s multiple comparison test and results were reported as significant if *P-*value was below 0.05. Phototoxicity SRB data of B16-F10, MeL, FM55 and N/TERT-1 was analyzed via student *t* test and significance was reported if *P-*value was below 0.05.

**Notes**

^⊥^The proliferation graphs are representative of single experiment that was performed thrice independently.

^ϕ^The data represent mean value ± SEM of three independent experiments with triplicates.

## Electronic supplementary material

Supplementary Information

## References

[1] Alexiades-Armenakas M (2006). Laser-mediated photodynamic therapy. Clin. Dermatol..

[2] Dolmans DE, Fukumura D, Jain RK (2003). Photodynamic therapy for cancer. Nat. Rev. Cancer.

[3] Yano S, Hirohara S, Obata M, Hagiya Y, Ogura S-I, Ikeda A, Kataoka H, Tanaka M, Joh T (2011). Current states and future views in photodynamic therapy. J. Photochem. Photobiol. C: Photochem.Rev..

[4] Celli JP, Spring BQ, Rizvi I, Evans CL, Samkoe KS, Verma S, Pogue BW, Hasan T (2010). Imaging and photodynamic therapy: mechanisms, monitoring, and optimization. Chem. Rev..

[5] Weishaupt KR, Gomer CJ, Dougherty TJ (1976). Identification of singlet oxygen as the cytotoxic agent in photoinactivation of a murine tumor. Cancer Res..

[6] Calzavara-Pinton PG, Venturini M, Sala R (2007). Photodynamic therapy: update 2006. Part 2: Clinical results. J. Eur. Acad. Dermatol. Venereol..

[7] Abels C (1997). Photodynamic therapy with 5-aminolaevulinic acid-induced porphyrins of an amelanotic melanoma *in vivo*. J Photochem. Photobiol. B.

[8] Wachowska M (2011). Aminolevulinic Acid (ALA) as a Prodrug in Photodynamic Therapy of Cancer. Molecule.

[9] Baldea I, Filip AG (2012). Photodynamic therapy in melanoma–an update. J. Physiol. Pharmacol..

[10] Barge J (2004). Killing efficacy of a new silicon phthalocyanine in human melanoma cells treated with photodynamic therapy by early activation of mitochondrion-mediated apoptosis. Exp. Dermatol..

[11] Szurko A (2003). Photodynamic effects of two water soluble porphyrins evaluated on human malignant melanoma cells *in vitro*. Acta. Biochim. Pol..

[12] Saczko J (2005). The influence of photodynamic therapy on apoptosis in human melanoma cell line. Folia. Histochem. Cytobiol..

[13] Ickowicz Schwartz D (2004). Differentiation-dependent photodynamic therapy regulated by porphobilinogen deaminase in B16 melanoma. Br. J. Cancer.

[14] Maduray K, Karsten A, Odhav B, Nyokong T (2011). *In vitro* toxicity testing of zinc tetrasulfophthalocyanines in fibroblast and keratinocyte cells for the treatment of melanoma cancer by photodynamic therapy. J. Photochem. Photobiol. B.

[15] Otake E (2010). Effect and mechanism of a new photodynamic therapy with glycoconjugated fullerene. Photochem. Photobiol..

[16] Ozler SA (1992). Photodynamic therapy of experimental subchoroidal melanoma using chloroaluminum sulfonated phthalocyanine. Arch. Ophthalmol..

[17] Hu L (2002). Photodynamic therapy of pigmented choroidal melanomas in rabbits. Zhonghua Yan Ke Za Zhi.

[18] Young LH (1996). Photodynamic therapy of pigmented choroidal melanomas using a liposomal preparation of benzoporphyrin derivative. Arch. Ophthalmol..

[19] Haddad R (1998). *In vitro* and *in vivo* effects of photodynamic therapy on murine malignant melanoma. Ann. Surg. Oncol..

[20] Haddad R (2000). Photodynamic therapy of murine colon cancer and melanoma using systemic aminolevulinic acid as a photosensitizer. Int. J. Surg. Investig..

[21] Rosenkranz AA (2013). Malignant melanoma and melanocortin 1 receptor. Biochemistry (Mosc).

[22] Slastnikova TA (2012). Modular nanotransporters: a multipurpose *in vivo* working platform for targeted drug delivery. Int. J. Nanomedicine..

[23] Donaldson MJ (2005). Primary treatment of choroidal amelanotic melanoma with photodynamic therapy. Clin. Experiment Ophthalmol..

[24] Soucek P, Cihelkova I (2006). Photodynamic therapy with verteporfin in subfoveal amelanotic choroidal melanoma (A controlled case). Neuro. Endocrinol. Lett..

[25] Barbazetto IA (2003). Treatment of choroidal melanoma using photodynamic therapy. Am. J. Ophthalmol..

[26] Panagopoulos JA (1989). Photodynamic therapy for experimental intraocular melanoma using chloroaluminum sulfonated phthalocyanine. Arch. Ophthalmol..

[27] Dabrowski JM (2011). Tissue uptake study and photodynamic therapy of melanoma-bearing mice with a nontoxic, effective chlorin. Chem. Med. Chem..

[28] Abrahamse H, Hamblin MR (2016). New photosensitizers for photodynamic therapy. Biochem. J..

[29] Bouamaied I, Stulz E (2009). Thieme Chemistry Journal Awardees - Where are They Now? Stabilisation of Porphyrins in Tetranucleotide-Bisporphyrin Arrays by Duplex Formation with Peptide Nucleic Acid. Synlett..

[30] Quan WD (2016). Retaining individualities: the photodynamics of self-ordering porphyrin assemblies. Chem. Commun. (Camb).

[31] Stulz E (2015). Porphyrin-modified DNA as Construction Material in Supramolecular Chemistry and Nano-architectonics. Chimia (Aarau).

[32] Motiei L (2014). Targeted protein surface sensors as a tool for analyzing small populations of proteins in biological mixtures. Angew. Chem. Int. Ed..

[33] Nissinkorn Y (2015). Sensing Protein Surfaces with Targeted Fluorescent Receptors. Chem. Eur. J..

[34] Peri-Naor R, Ilani T, Motiei L, Margulies D (2015). Protein-Protein Communication and Enzyme Activation Mediated by a Synthetic Chemical Transducer. J. Am. Chem. Soc..

[35] Hamblin MR, Miller JL, Hasan T (1996). Effect of charge on the interaction of site-specific photoimmunoconjugates with human ovarian cancer cells. Cancer. Res..

[36] Oseroff AR (1986). Antibody-targeted photolysis: selective photodestruction of human T-cell leukemia cells using monoclonal antibody-chlorin e6 conjugates. Proc. Natl. Acad. Sci. (USA).

[37] Ogura S (2005). Localization of poly-L-lysine-photosensitizer conjugate in nucleus. J. Control Release.

[38] Choi Y, Weissleder R, Tung CH (2006). Selective antitumor effect of novel protease-mediated photodynamic agent. Cancer Res..

[39] Abu-Yousif AO (2012). Epidermal growth factor receptor-targeted photosensitizer selectively inhibits EGFR signaling and induces targeted phototoxicity in ovarian cancer cells. Cancer Lett..

[40] You H (2015). Pheophorbide – a conjugates with cancer-targeting moieties for targeted photodynamic cancer therapy. Bioorg. Med. Chem..

[41] Kascakova S (2014). Somatostatin analogues for receptor targeted photodynamic therapy. PLoS One..

[42] Hamblin MR (1994). Photosensitizer targeting in photodynamic therapy. I. Conjugates of haematoporphyrin with albumin and transferrin. J. Photochem. photobiol. B..

[43] Hamblin MR, Newman EL (1994). Photosensitizer targeting in photodynamic therapy. II. Conjugates of haematoporphyrin with serum lipoproteins. J. Photochem. photobiol. B..

[44] Park SY (2011). A smart polysaccharide/drug conjugate for photodynamic therapy. Angew. Chem. Int. Ed..

[45] Yuan Q (2013). Targeted bioimaging and photodynamic therapy nanoplatform using an aptamer-guided G-quadruplex DNA carrier and near-infrared light. Angew. Chem. Int. Ed..

[46] Cheng L (2014). Functional nanomaterials for phototherapies of cancer. Chem. Rev..

[47] Jain RK (2001). Delivery of molecular and cellular medicine to solid tumors. Adv. Drug. Deliv. Rev..

[48] Friedrich SW (2002). Antibody-directed effector cell therapy of tumors: analysis and optimization using a physiologically based pharmacokinetic model. Neoplasia.

[49] Froidevaux S (2004). A gallium-labeled DOTA-alpha-melanocyte- stimulating hormone analog for PET imaging of melanoma metastases. J. Nucl. Med..

[50] Froidevaux S, Calame-Christe M, Tanner H, Sumanovski L, Eberle AN (2002). A novel DOTA-α-melanocytestimulating hormone analog for metastatic melanoma diagnosis. J. Nucl. Med..

[51] Siegrist W, Solca F, Stutz S, Giuffr. L, Carrel S, Girard J, Eberle AN (1989). Characterization of receptors for α-melanocyte-stimulating hormone on human melanoma cells. Cancer Res..

[52] Eberle AN (2010). MSH radiopeptides for targeting melanoma metastases. Adv. Exp. Med. Biol..

[53] Siegrist W, Girard J, Eberle AN (1991). Quantification of MSH receptors on mouse melanoma tissue by receptor autoradiography. J. Recept. Res..

[54] Eberle, A. N. The Melanotropins; Chemistry, Physiology and Mechanisms of Action; Karger: Basel (1988).

[55] Nelson JS (1988). Photodynamic therapy of human malignant melanoma xenografts in athymic nude mice. J. Natl. Cancer Inst..

[56] Huang Y-Y (2013). Melanoma resistance to photodynamic therapy: new insights. Biol Chem..

[57] Kalluri H, Banga AK (2011). Transdermal delivery of proteins. AAPS Pharm. Sci. Tech..

[58] Debele TA (2015). Drug Carrier for Photodynamic Cancer Therapy. Int. J. Mol. Sci..

[59] Mustafa F, Jaafar M (2013). Comparison of wavelength-dependent penetration depths of lasers in different types of skin in photodynamic therapy. Indian J Phys..

[60] Bigliardi, P. L. *et al*. Multi-spectral calibrated light source for long term cell culture study. Provisional SG Patent Number: 10201501747P, March 6 (2015).

[61] Donatien PD (1992). The expression of functional MSH receptors on cultured human melanocytes. Arch. Dermatol. Res..

[62] Sregrist W, Eberle AN (1986). insitu-melanin assay for MSH using B16 melanoma cells in culture. Anal. Biochem..

[63] Eberle AN, Rout B, Bigliardi-Qi M, Bigliardi PL (2017). Synthetic Peptide Drugs for Targeting Skin Cancer: Malignant Melanoma and Melanotic Lesions. Curr. Med. Chem..

[64] Fitzpatrick TB, Breathnach AS (1963). The epidermal melanin unit system. Dermatol. Wochenschr..

[65] Costin GE, Hearing VJ (2007). Human skin pigmentation: melanocytes modulate skin color in response to stress. Faseb. J..

[66] Sriram G (2015). Fibroblast heterogeneity and its implications for engineering organotypic skin models *in vitro*. Eur. J. Cell Biol..

[67] Xiong Z-M (2017). Anti-Aging Potentials of Methylene Blue for Human Skin Longevity. Sci. Rep..

