# Supplementary material for: Specific Targeting of Melanotic Cells with Peptide Ligated Photosensitizers for Photodynamic Therapy
Source: Sci Rep. 2017 Nov 16;7:15750. doi: 10.1038/s41598-017-15142-w (PMC5691209; doi:10.1038/s41598-017-15142-w)
Supplement: Supplementary file 1 — Supplementary Information [file 41598_2017_15142_MOESM1_ESM.pdf]

Supplementary Information

Specific Targeting of Melanotic Cells with Peptide Ligated Photosensitizers for Photodynamic Therapy

Paul Lorenz Bigliardi[a,b,c,d, †], Bhimsen Rout[a,b,e,†,*], Aakanksha Pant[a, †], Viknish Krishnan-Kutty[a], Alex N. Eberle[f], Ramasamy Srinivas[a], Brendan Adrian Burkett[e,*], Mei Bigliardi-Qi[a,b,*]

a) Experimental Dermatology Group, Institute of Medical Biology, A*STAR, 8-Biomedical Grove, Singapore-138648.
b) Clinical Research Unit for Skin, Allegy and Regeneration, IMB, A*STAR, Singapore
c) National University Hospital Singapore
d) NUS Yong Loo Lin School of Medicine, Singapore.
e) Department of Organic Chemistry, Institute of Chemical and Engineering Sciences, A*STAR, 8-Biomedical Grove, Singapore-138665.
f) Department of Biomedicine, University of Basel, Switzerland.

[†] The authors contributed equally to the manuscript as first author.
* The authors contributed equally to the manuscript as corresponding author.

Email: mei.bigliardi@imb.a-star.edu.sg; brendan_burkett@ices.a-star.edu.sg; bhimsen.rout@imb.a-star.edu.sg

Contents

1. Materials and Methods……………………………………………………………………S3

2. Synthetic Procedure ……………………..………………………..…...…………………………….S4

a. Synthesis of HPPH-NAP……………….………………………..……….……....…..S4

b. Synthesis of Linker **2**..………S5

c. Synthesis of MB-Linker **3**…………………….……………..……………………...……...S5

d. Synthesis of MB-Linker-acid **4**…………………….……………..……………………...……...S6

e. Synthesis of NAP-MB…………..…………………….….……………………………………S6

3. Cell proliferation/Growth assays………………………………………………………….S7

a. Determination of working concentration of various synthetic peptide-photosensitizer constructs; General procedure for dark proliferation…………………………………………………….….S7

b. General procedure for proliferation experiments with synthetic peptide-photosensitizer constructs and light irradiation……………………………………………………………………...….S8

c. Light control experiment…………………………...……………………..………………….S9

4. Competition assay…………………………………………………………………………….S10

1. Materials and Methods:

Chemicals and anhydrous solvents were obtained from Sigma Aldrich and were used without further purification. Spectroscopic grade solvents were purchased from Sigma Aldrich. Peptide sequences were purchased from Hisunny Chemicals Co. Ltd. Anhydrous solvents were transferred using oven-dried syringes. The flash column was used to purify all synthetic intermediates. The purification of peptides was performed by preparative reverse phase HPLC using Jupiter C12 Proteos 90Å RP-HPLC column employing a binary gradient of solution A (0.1% TFA in H_2O) and solution B (0.1% TFA in acetonitrile). The purity of peptides was ascertained by analytical reverse phase HPLC using Jupiter C4 Proteos 90Å RP-HPLC column employing a binary gradient of solution A (0.1% TFA in H_2O) and solution B (0.1% TFA in acetonitrile). The HPLC purified fractions were freeze dried using a Labonco lyophilizer at -60 ^{o}C and 0.01 mbar vacuum. The 1H NMR spectra of all compounds were recorded on a Bruker 400 MHz NMR spectrometer. Mass spectra were analyzed by Water LC-micro spectrometer using H_2O/acetonitrile (1:1, v:v). Absorption spectrum measurement was performed on a Varian technology international UV spectrometer using 96-well plates.

Cells were seeded at densities of 60,000 cells per well in Dulbecco's Modified Eagle's medium (DMEM) without phenol red in Nunc 6-well tissue culture plates. α-MSH was obtained from Sigma-Aldrich and Abcam. 3-Isobutyl-1-methylxanthine (IBMX) was purchased from Sigma Aldrich and was used as an internal standard to increases c-AMP levels in cells leading to increased melanin production. Melanin absorbance assay were done using Spectra Max MS microplate/cuvette reader at optical density of 475 nm.

2. Synthetic Procedure:

a) Synthesis of HPPH-NAP:

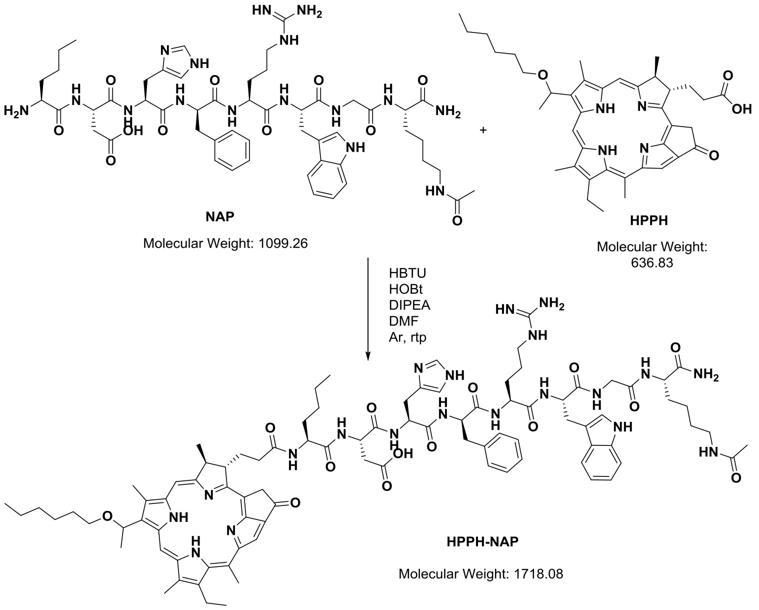

Procedure: To a stirred solution of HPPH (62.4 mg, 98 µmol) in 7 mL of DMF was added DIPEA (34 µL, 195.9 µmol) followed by HBTU (37.2 mg, 98 µmol) and HOBt (13.2 mg, 98 µmol) at room temperature. After 10 min, peptide NAP (140 mg, 127 µmol; dissolved in 1 ml of DMF) was added dropwise. The reaction mixture was stirred for 18 h. The solvent was evaporated at high vacuum at 40 ^{o}C. The crude reaction mass was purified by preparative HPLC using a detector at 406 nm, 220 nm, 254 nm and the following gradient of 0.1% TFA in H_2O as solvent A and 0.1% TFA in acetonitrile as solvent B.

Time (min)	Solvent B (%)	Flow rate (mL/min)
0	20	5
2	20	5
5	90	5
16.5	90	5
18.5	20	5
20	20	5

The purity of the fractions was ascertained by analytical HPLC using same gradient of solvents. The fractions of similar purity were combined and lyophilized. The yield of HPPH-NAP was 50.4 mg (30%); HPLC purity = 95%. MS (ESI+): m/z (%) = 1718.74 (5) $[M]^{+}$, 860.3 (30) $[M+2H]^{+2}$, 573.91 (100) $[M+3H]^{+3}$.

b) **Synthesis of linker 2:**

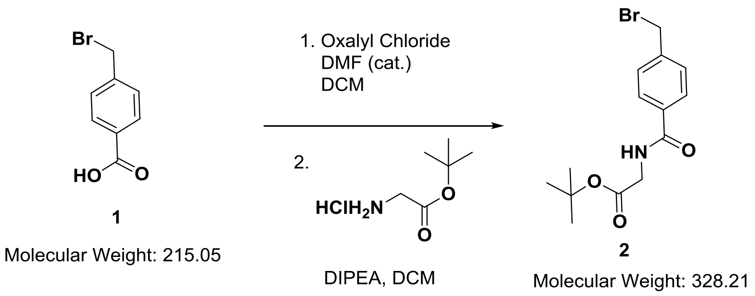

Procedure: To a stirred solution of 4-bromomethyl benzoic acid **1** (100 mg, 0.465 mmol) in 4 mL dry dichloromethane (DCM) were added oxaloyl chloride (413 µL, 4.65 mmol) and 2 drops (Cat.) of di-methylformamide (DMF) at room temperature. The reaction mixture was stirred overnight and the solvent was evaporated using a rotary evaporator and vacuum at room temperature. The yellow solid was dried under high vacuum for 3 h and dissolved in 4 mL dry DCM. N, N-Diisopropylethylamine (DIPEA) (243 µL, 1.395 mmol) was added to the above solution, followed by the addition of t-butyl glycine (85.7 mg, 0.515 mmol). The reaction mixture was stirred at room temperature for 6 h. The solvent was evaporated and the crude reaction mixture was purified by silica gel column chromatog-raphy using methanol:DCM (1:99, v:v) to furnish **2** in 60% yield (91 mg).

^{1}H NMR (400 MHz, chloroform-d) δ 7.88 – 7.77 (m, 2H), 7.54 – 7.43 (m, 2H), 4.63 (s, 2H), 4.16 (d, *J* = 4.9 Hz, 2H), 1.53 (s, 9H). MS (ESI$^+$): m/z (%) = 228.21 (25) [M-^{t}Bu-CO_2]$^+$

c) **Synthesis of MB-linker 3:**

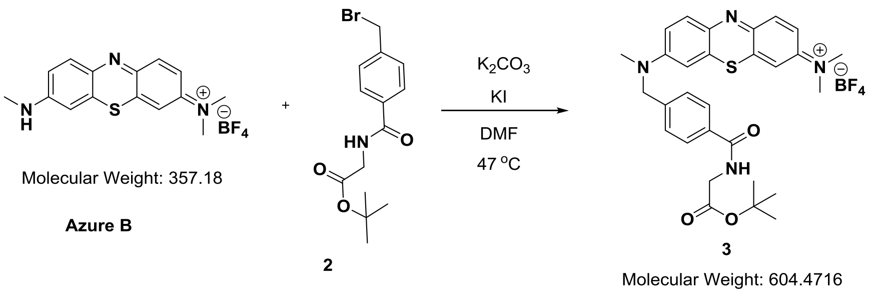

Procedure: To a stirred solution of Azure B (150 mg, 0.418 mmol) in anhydrous DMF (4 mL) were added K_2CO_3 (110 mg, 0.836 mmol) and **2** (137 mg, 0.418 mmol) under argon at 47 ^oC. To the above mixture KI (60 mg, 0.418 mmol) were added. After 1.5 h of heating, **2** (137 mg, 0.418 mmol) was added. After 3 h of heating, an additional amount of **2** (137 mg, 0.418 mmol) was added. After 5 h,

the DMF solvent was evaporated at 43 °C using high vacuum and the crude reaction mass was subjected to silica gel column chromatography using MeOH:DCM (7:93, v:v) to furnish **3** in 30% yield (50 mg).

^{1}H NMR (400 MHz, chloroform-d) δ 7.72 (d, *J* = 8.4 Hz, 4H), 7.28 – 6.99 (m, 6H), 4.77 (s, 2H), 3.97 (d, *J* = 5.1 Hz, 2H), 3.70 – 3.50 (m, 3H), 3.26 (s, 6H), 1.34 (s, 9H). MS (ESI+): m/z (%) = 517.43 (100) $[M\text{-}BF_4]^+$, 461.36 (25) $[M+H\text{-}CO_2\text{-}BF_4]^+$.

d) **Synthesis of MB-linker 1-acid 4:**

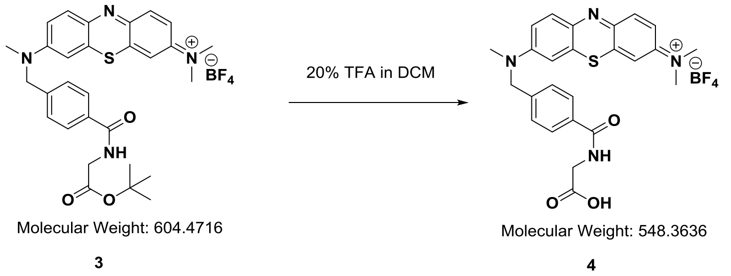

Procedure: To a stirred solution of **3** (24 mg, 0.0529 mmol) in 0.8 mL of DCM, 0.2 mL of a solution of trifluoroacetic acid were added dropwise at room temperature. The reaction mixture was stirred for 3 h and the consumption of t-butyl ester monitored by analytical HPLC. The solvent was evaporated using rotary evaporator and vacuum. The crude acid **4** has taken further without purification. MS (ESI+): m/z (%) = 461.37 (100) $[M\text{-}CO_2\text{-}BF_4]^+$.

e) **Synthesis of NAP- MB:**

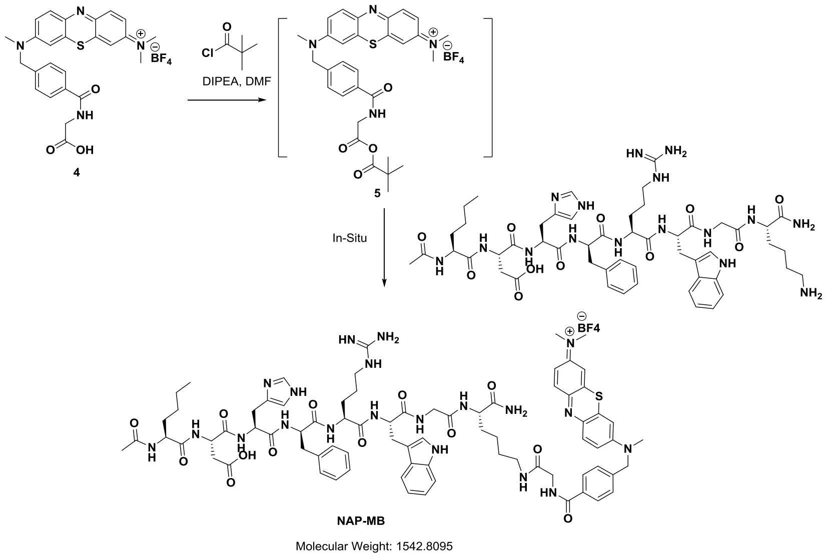

Procedure: To a stirred solution of **4** (29 mg, 0.0529 mmol) in 1 mL of DMF was added DIPEA (27.6 µL, 0.1587 mmol) followed by pivaloyl chloride (7 µL, 0.0582 mmol) at room temperature. After 1.5

h, peptide (58 mg, 0.0529 mmol; dissolved in 1 ml of DMF) was added dropwise. The reaction mixture was stirred for 3 h. The solvent was evaporated at high vacuum at 40 ^{o}C. The crude reaction mass was purified by preparative HPLC using a detector at 640 nm, 210 nm, 254 nm and the following gradient of 0.1% TFA in H_2O as solvent A and 0.1% TFA in acetonitrile as solvent B.

Time (min)	Solvent B (%)	Flow rate (mL/min)
0	20	5
2	30	5
20	85	5
22	85	5
23.5	20	5
25	20	5

The purity of freeze-dried sample (12 mg) was 80% and was further purified using detector at 640 nm, 210 nm, 254 nm and following gradient.

Time (min)	Solvent B (%)	Flow rate (mL/min)
0	30	5
2	30	5
28	70	5
29.5	70	5
31	30	5
23	30	5

The purity of the fractions was ascertained by analytical HPLC using same gradient of solvents at 640 nm. The fractions of similar purity were combined and lyophilized. Weight of NAP-MB = 8 mg; purity (HPLC) = 100%. MS (ESI+): m/z (%) = 1542.36 (5) $[M-BF_4]^+$, 772.09 (50) $[M+2H-BF_4]^{+2}$, 515.25 (100) $[M+3H-BF_4]^{+3}$.

3. **Cell Proliferation/ Growth Assay:**

a) **Determination of working concentrations of various synthetic peptide-photosensitizer constructs; General procedure for dark proliferation:** B16-F10 cells were treated with 10 µM, 1 µM, 500 nM, 250 nM, and 100 nM concentrations of HPPH-NAP (figure S1a) or NAP-MB (figure S1b) for 4 h and allowed to proliferate at 37 ^{o}C without light exposure. Cells were imaged at an interval of 1 h using the incucyte microscope in dark. HPPH-NAP, NAP-MB have similar proliferation as media and were found nontoxic at 5 µM, 10 µM and 1 µM respectively in dark. This was further supported by the

images of the cell morphologies. Hence, these concentrations were considered as optimum for respective synthetic peptide-photosensitizer constructs under light mediated cytotoxic measurements.

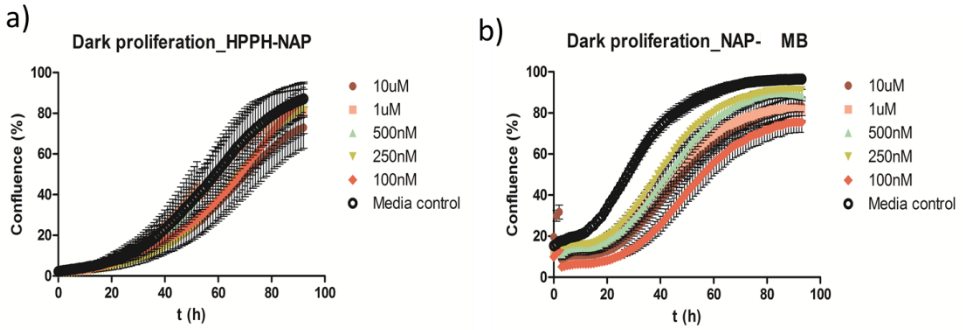

Figure S1: Proliferation graphs of B16-F10 cells with different concentrations of a) HPPH-NAP, b) NAP-MB and in the absence of light.

b) General procedure for proliferation experiments with synthetic peptide-photosensitizer constructs and light irradiation: B16-F10 melanoma cells / N-TERT-1 keratinocytes were seeded in a 96-black Perkin-Elmer well plate in DMEM medium without phenol red and kept for overnight at 37 ^{o}C. The cells were incubated with the synthetic peptide-photosensitizer constructs at the optimized concentrations in the dark for 4 h and then washed twice with 1X PBS (200 mL). At this stage, DMEM media was changed with fresh DMEM media (300 µL). Cells were irradiated at 660 nm wavelength using Incucyte-LED system (principle described in main text, methods). The light intensity was kept constant at 0.10 mW/cm^2 for different time intervals. Cell proliferation was monitored by taking incucyte image at an interval of 1h to obtain change in cell density (i.e. confluency). The images obtained after 24 h were used for comparative analysis of combinatorial effect of light and synthetic peptide-photosensitizer constructs on cell morphologies.

For cytotoxic study of HPPH-NAP (5 µM) and NAP-MB (10 µM) under light at 660 nm, B16-F10 mouse melanoma cells were seeded at a density of 4000 cells per well and cell density growth at each hour were shown in proliferation curve in the main text figure 5a, and figure 6a respectively. Similarly, images taken after 24 h were used for comparative analysis of light effect on cell morphologies as shown in figure 5 (c-d), and figure 6 (c-d) respectively in the main text. N/TERT-1 keratinocytes were grown to a confluence of 75-85% before HPPH-NAP (5 µM) or NAP- MB (10 µM) incubation. Cell density growth at each hour were shown in proliferation curve in the main text figure 5b, and figure 6b respectively for HPPH-NAP (5 µM) and NAP-MB (10 µM). Similarly, images taken after 24 h were

used for comparative analysis of light effect on cell morphologies is shown in figure 5 (e-f) and figure 6 (e-f) respectively in the main text.

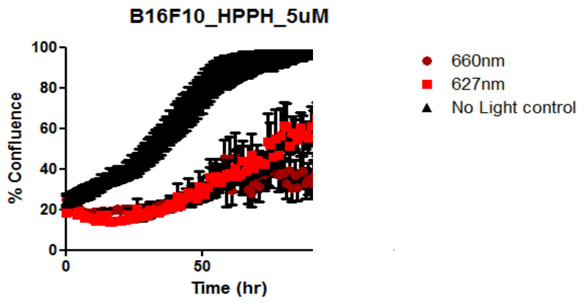

Figure S2: Cell proliferation curve of B16-F10 by 5 µM HPPH with 660 nm (red), 627 nm (brown) and no light control (black).

a) **Light control experiment:** To see the effect of light (660 nm) alone on cell growth, B16-F10 and N/TERT-1 cells were exposed to light at wavelength 660 nm continuously for 24 h and cell proliferation was monitored (Figure S3) via incucyte.

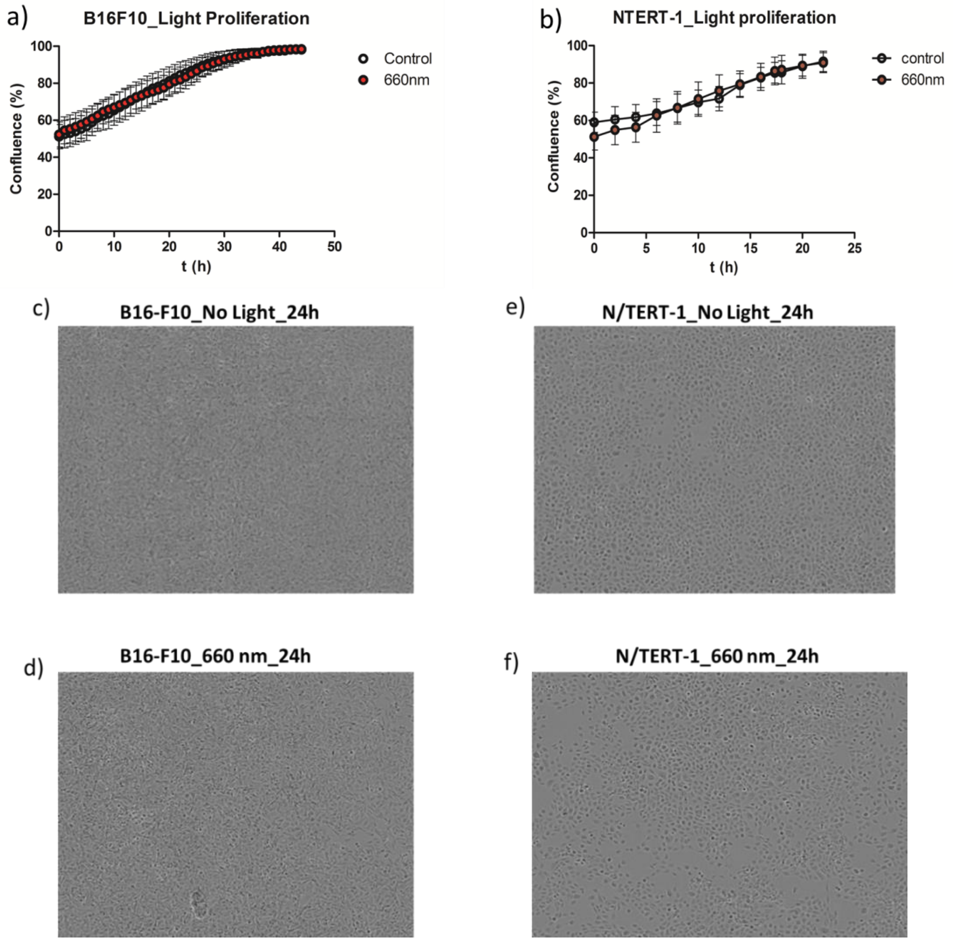

Figure S3: Proliferation effect of red light (a) on B16-F10 cells for 24 h (660 nm) and (b) on N/TERT-1 cells and (c) and (d) as corresponding images after light exposure of 24h along with no light exposed controls (e, f).

Both B16-F10 mouse melanoma cells and human keratinocytes N/TERT-1 were seeded at cell densities to achieve similar confluence after overnight incubation. Cell density growth at each hour was shown in proliferation curve in the figure S3a, and figure S3b for B16-F10 and N/TERT-1, respectively. This indicates light has no effect on the proliferation of both melanoma cells and keratinocytes. Incucyte images were taken after 24 h used for comparative analysis of light effect on cell morphologies in figure S3 (c, d) for B16-F10 and figure S3 (e, f) for N/TERT-1 designates intact or healthy cell morphologies even after 24 h light irradiation.

4. Competition assays:

B16F10 melanoma cells were pre-treated with NAP (250 µM) at room temperature (RT) or 4 ^{o}C for 30 mins. Subsequently, cells were incubated in a solution of NAP-MB (1 µM) plus competitor (NAP; 250 µM) for another 30 mins at RT or 4 ^{o}C and subjected to flow cytometry analysis acquisition.
